# Preparation and Characterization of Fly Ash Coated with Zinc Oxide Nanocomposites

**DOI:** 10.3390/ma12213550

**Published:** 2019-10-29

**Authors:** Caili Wang, Jing Wang, Liqi Bai, Runquan Yang, Huaifa Wang

**Affiliations:** College of Mining Engineering, Taiyuan University of Technology, Taiyuan 030024, China; wj92ok@126.com (J.W.); bailiqi@link.tyut.edu.cn (L.B.); yangrunquan@tyut.edu.cn (R.Y.);

**Keywords:** fly ash, zinc oxide, whiteness, nanocomposites

## Abstract

Calcined fly ash (CFA) was first obtained by calcining fly ash (FA) at 815 °C for two hours. Then, composite powders of CFA coated with zinc oxide nanoparticles (ZnO/CFA, ZCFA) were prepared by heterogeneous nucleation method. The samples were characterized by X-ray diffraction (XRD), Fourier transform infrared (FTIR) spectroscopy, Scanning electronic microscopy (SEM), Whiteness, and Brunauer-Emmett-Teller specific surface area (BET). Effects of pH value, reaction temperature and time, coating amount, solid-to-liquid ratio, the coating agent concentrations, and dropping speed on the whiteness of ZCFA powders were studied. It was shown that after coated with ZnO particles, the whiteness of CFA was increased from 27.0 to 62.6%, and the specific surface area was increased from 5.80 to 14.61 m^2^/g. Needle ZnO with the average grain size of 46 nm was deposited on the surface of CFA. Si–O–Zn–OH bonds were formed.

## 1. Introduction

Recently, more and more attention has been paid to the research of fly ash (FA) for that it has taken up large amounts of land and tends to cause pollution to the environment [1]. Surface treatment and application of FA has been considered as the high value-added way to solve this problem [2]. FA has been used in several areas, such as construction and building materials [3], cement and concrete applications [4], photocatalysis [5], agriculture [6], polymer [7], sewage treatment [8], and geopolymers [9,10], among which, the application of FA as filler in polymer has been considered as the most high value-added field [11]. The reason is that FA not only supplements the bulk volume of the polymer itself, but also facilitates compounding and processing when custom-shaped products are fabricated [11]. However, the use of FA as filler is still not wide-spread. The main reasons are as follows [12]: (1) The low whiteness value of FA resulting in an undesirable appearance to the final product, (2) the high mohs hardness of FA resulting in high abrasion of the processing equipments, and (3) the weak interfacial bonding between untreated FA and polymer owing to the low friction of the FA surface resulting in low mechanical properties of composite materials. Yang deposited nano calcium carbonate on the surface of FA to prepare composite materials and fill it in PP. This study solved the above-mentioned three problems to some extent and opened the door for the preparation of composite materials [12]. 

Nowadays, not only the mechanical properties but also the flame retardant properties of polymer are required in many applications. Polyamide 6 (PA6) is widely used in electron and electric industries because of its excellent properties such as outstanding mechanical properties, high resistance against abrasion, easy processability, and good chemical stability [13]. However, there are two obvious disadvantages related to low heat distortion temperature (about 70 °C) and a low oxygen index (21%) of PA6 [14], which affects its application. Consequently, improving the fire retardant behavior and heat distortion temperature of PA6 is a major challenge for extending their use to most applications [14]. Halogenated additives have been used as flame retardant additives to increase the fire retardant behavior of polymers [15]. However, they are being phased out for their adverse effects on the environment. Wang et al. coated aluminum silicate on fly ash and filled it in PA6, resulting in an increase of the distortion temperature, mechanical properties, and whiteness of PA6 [13]. In view of this, if we can deposit a new nano material which has low mohs hardness, high whiteness, and flame retardant properties on the surface of FA and fill it in polymer, not only the three problems mentioned above can be solved, but also the flame retardant properties of polymer composites could be enhanced. 

Mg(OH)_2_, Al(OH)_3_, and zinc oxide (ZnO) have high whiteness and low mohs hardness and are widely applied as inorganic flame retardant in filling polymer [16,17,18]. However, nano Mg(OH)_2_, Al(OH)_3_, and ZnO have poor dispersion because of their small size and large specific surface area. Mg(OH)_2_ and Al(OH)_3_ as inorganic flame retardant have been studied by many experts while little attention has been paid to ZnO. 

Micron size FA powder has an advantage to easily disperse in filling polymer. According to the thought of particle design, if nano zinc oxide was deposited on the surface of FA, not only the fly ash-based composite can be used in filling polymer to obtain higher flame retardant and mechanical properties, high whiteness, and low mohs hardness, but also the dispersion of nano zinc oxide can be improved. 

The heterogeneous nucleation method is an efficient synthetic method for composite materials with uniform shape and size [19]. In this paper, the preparation of calcined fly ash (CFA) coated with zinc oxide nanoparticles (ZnO/CFA, ZCFA) with a heterogeneous nucleation method taking CFA as the substrate was reported. The effect of different experimental parameters including pH value, reaction temperature, coating amount, solid-to-liquid ratio, dropping speed, reaction time, and the concentration of NaOH and ZnSO_4_ on the whiteness of ZCFA powders was studied and the prepared ZCFA was characterized.

## 2. Materials and Methods

### 2.1. Materials

The fly ash particles with a whiteness of 13.7% used as the substrate, was obtained from ShangHai GeRunYa Nano-Material Co. Ltd. (Shanghai, China) The chemical composition consisted of 54.7 wt% SiO_2_, 29.79 wt% Al_2_O_3_, 4.06 wt% TFe_2_O_3_, 1.25 wt% TiO_2_, and 3.30 wt% CaO in which SiO_2_ and Al_2_O_3_ accounted for nearly 84.49%. The loss on ignition is 3.26%. The zinc sulfate (purity 98%) used in the experiment was purchased from Tianjin Zhiyuan chemical reagent manufacturing Co. (Tianjin, China). The sodium hydroxide (purity 98%) used in the experiment was purchased from Tianjin Hengxing chemical reagent manufacturing Co. (Tianjin, China). All chemicals were of analytical grade and used without further purification. 

### 2.2. The Composite Preparation

The FA was calcined in the muffle furnace at 815 °C for 2 h to prepare CFA. The purpose is to remove the carbon. The whiteness of FA was increased from 13.7 to 27.0% after calcination. The preparation of ZCFA was carried out the following way: Certain amounts of CFA powder were first dispersed in distilled water and stirred for 20 min at a certain temperature (below 100 °C). The zinc sulfate and sodium hydroxide solution were introduced to the agitated dispersion at a certain speed, while pH was adjusted through addition of sodium hydroxide, and then stirred for a further certain time. After some minutes, the precipitate was washed with distilled water several times in order to remove the residual ions. Finally, the precipitate was dried at 105 °C for 16 h and ZCFA powders were obtained. The influence of pH value, reaction temperature, coating amount, solid-to-liquid ratio, dropping speed, reaction time, and the concentration of NaOH and ZnSO_4_ on the whiteness of the ZCFA powders was studied.

### 2.3. Physical Property Measurement

The whiteness of FA, CFA, and ZCFA was examined by DN-B type whiteness analyzer (Hangzhou High Tech Automation Instrument Co., Ltd, HangZhou, China). Scanning electronic microscopy (SEM) micrographs of FA, CFA, and ZCFA powders were obtained with a JSM-7001F electron microscope (Japan Electron Optics Laboratory Co., LTD, Akishima, Japan). The specific surface areas of FA, CFA, and ZCFA powders were measured by ST-2000 nitrogen sorption isotherm (Beijing Beifen Instrument Technology Co., Ltd., Beijing, China) measurement. X-ray diffraction (XRD) patterns of FA, CFA, and ZCFA powders were obtained by a MiniFlex600 X-ray diffractometer (Japan Rigaku Co., Ltd., Akishima, Japan) (Cu X-ray tube, 0.5°/min scan rate, 50 kV, 200 mA). Fourier transform infrared spectroscopy (FTIR) spectra of KBr disks were undertaken on a TENSOR27 spectrometer (German Bruker Co., Ltd., Karlsruhe, German). The zeta potential values of CFA and Zn(OH)_2_ under different pH values were detected with a JS94H zeta potential meter (Shanghai Zhongchen Digital Technology Co., Ltd., Shanghai, China).

## 3. Results and Discussion

### 3.1. Effects of Processing Parameters on ZCFA Whiteness and Morphology

**Figure 1:**
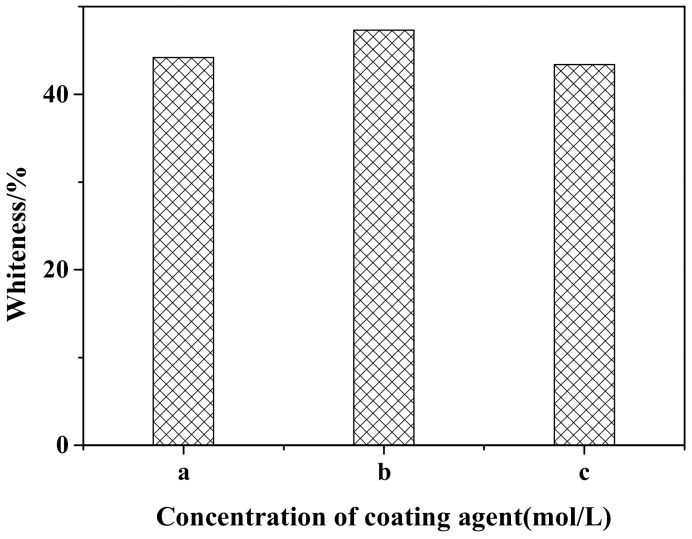
Whiteness of calcined fly ash coated with zinc oxide nanoparticles (ZCFA) with different coating agents concentrations: (**a**) 0.2 mol/L NaOH and 0.10 mol/L ZnSO_4_; (**b**) 0.3 mol/L NaOH and 0.15 mol/L ZnSO_4_; (**c**) 0.4 mol/L NaOH and 0.20 mol/L ZnSO_4._

Figure 1 shows the effect of different coating agent concentrations on the whiteness of ZCFA powders. It can be observed that when the concentrations of the NaOH and ZnSO_4_ solutions are 0.2 and 0.1 mol/L, respectively, the ions concentration in the solution is relatively low. The generated precipitation layer cannot completely cover the surface of CFA due to the lack of driving force, so the prepared ZCFA has low whiteness. When the concentrations of the NaOH and ZnSO_4_ solutions are 0.30 and 0.15 mol/L, respectively, the ions concentration in the solution and the driving force are appropriate and enough crystal nuclei are precipitated. The ZCFA powder has high whiteness. When the concentrations of the NaOH and ZnSO_4_ solutions are 0.4 and 0.2 mol/L, respectively, as the concentration of coating agent increases, the impetus of the reaction process increases, and the precipitation generated is prone to agglomeration. Therefore, the optimal concentrations of the NaOH solution and the ZnSO_4_ solution are 0.3 and 0.15 mol/L, respectively.

**Figure 2:**
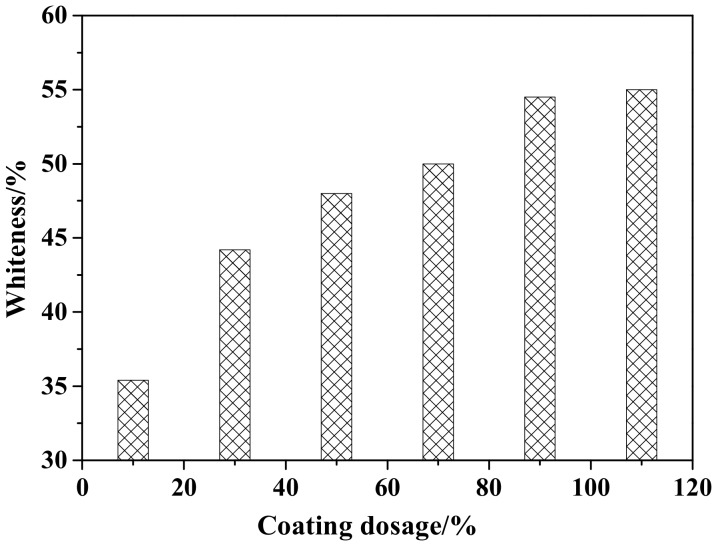
Whiteness of ZCFA powder with different coating dosages.

Figure 2 shows the whiteness of ZCFA under different coating amounts. It can be observed that the whiteness of ZCFA increases with the increase of the coating amount. When the coating amount is 90%, the ZCFA powder has the highest whiteness. By increasing the coating amount, the whiteness values did not have a greater change, so the best coating amount is 90%.

**Figure 3:**
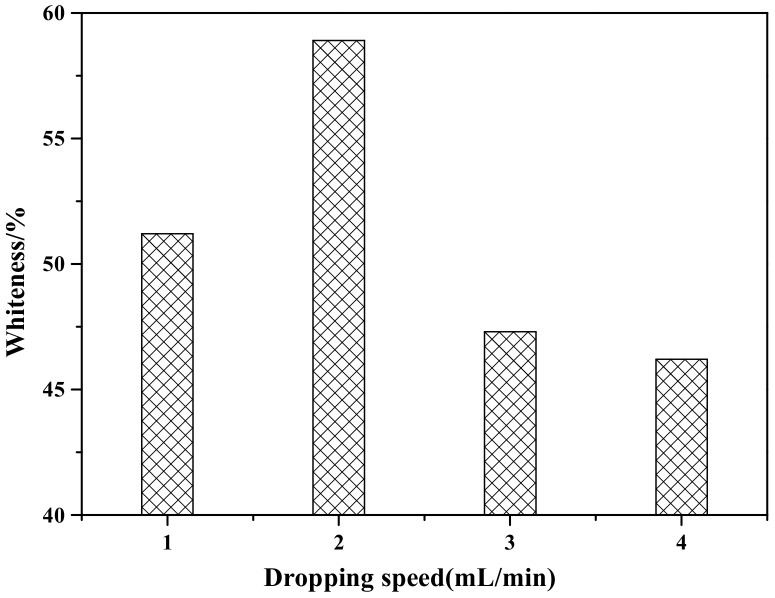
The effect of dropping speed of coating agent on whiteness of ZCFA powder.

Figure 3 shows the influence of the dropping speed of the coating agents (ZnSO_4_ and NaOH) on the whiteness of ZCFA composite powder. According to Figure 3, when the dropping speed of the coating agent is 2 mL/min, the prepared ZCFA has the highest whiteness. If the dropping speed is too slow, the ions concentration in the solution is too small, the reaction driving force is insufficient, and the particle size of precipitated crystal nucleus is smaller than the critical radius of crystal nucleus, and will be reintegrated into the solution. If the dropping speed of the coating agent is too fast, the hydrolysis reaction speed is fast and the ions concentration in the solution is high, which easily causes uneven coating. In addition, the newly generated Zn(OH)_2_ precipitation has large surface energy and activity, which will cause serious agglomeration. Therefore, the optimal dropping speed of the coating agent is 2 mL/min.

**Figure 4:**
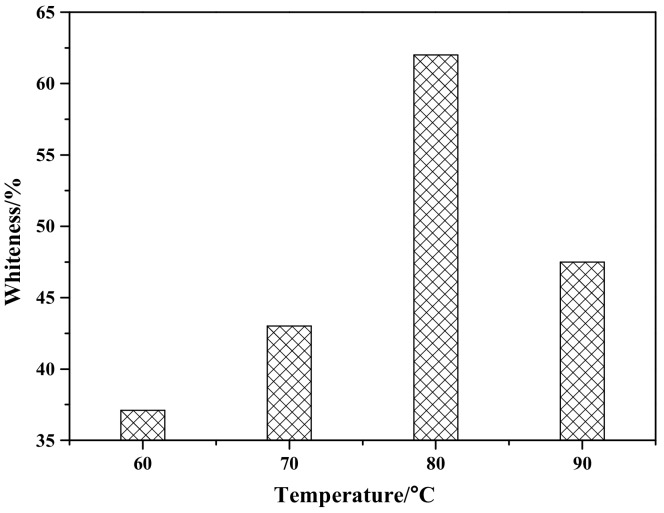
The effect of reaction temperature on whiteness of ZCFA powder.

**Figure 5:**
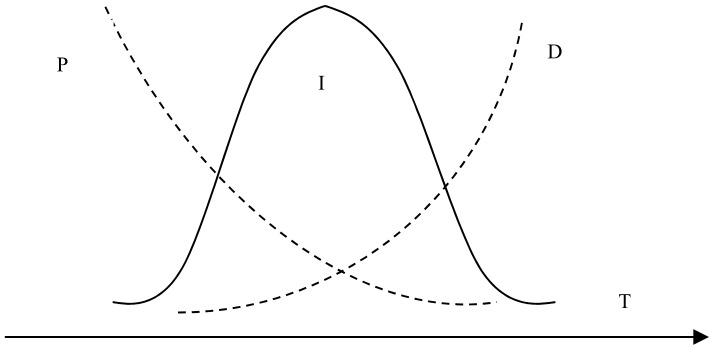
The relationship between nucleation rate and temperature.

Figure 4 shows the effect of reaction temperature on the whiteness of ZCFA composite powders. According to Figure 4, the whiteness value of the composite powder reached the highest point at 80 °C, which was 62.6%. When the reaction temperature is too low, the ions activation energy involved in the reaction is low, the Brownian motion is slow, the probability of ions collision is low, the diameter of Zn(OH)_2_ particles generated is large, and the contacting chance of Zn(OH)_2_ precipitation with CFA is reduced, which is not conducive to the deposition coating on the surface of CFA. With the increase of reaction temperature, the whiteness of ZCFA increased. The phenomenon can be explained by the Volmer nucleation rate formula. With the increase of temperature, Brownian motion in the solution became intense, the collision probability between particles increased, the reaction rate was improved, and a large number of Zn(OH)_2_ particles were generated and coated on the surface of CFA in a short time. In the process of heterogeneous nucleation, from Equations (1) and (2) [20], it can be seen that the critical radius of nucleation is determined by the degree of supercooling. The higher the reaction temperature is, the smaller the degree of supercooling is, and the larger the critical radius is, the more difficult the nucleation is. According to Equations (3)–(5), if *T* increases, *D* increases, the crystals are easy to precipitate. If *T* decreases, the particle movement resistance will increase and the particle movement rate in solution will decrease, which is not conducive to the nucleation process. It can be seen from Figure 5 that when *P* and *D* curves intersect, the nucleation rate is the largest and the optimal reaction temperature *T* exists. Therefore, the reaction temperature should not be too high or too low. That is, when the reaction temperature is 80 °C, the nucleation rate is the highest, and a large amount of Zn(OH)_2_ precipitation will be formed on the surface of CFA to form a dense coating layer and improve the whiteness of composite powders.

(1) $$r^{*}=\frac{2\sigma .T_{m}}{L_{m}.\Delta_{T}},$$

(2) $$\Delta_{T}=T_{m}-T,$$

(3) I=DP,

(4) $$D=exp(-\frac{\Delta G_{a}}{KT}),$$

(5) $$P=n_{0}\cdot q_{0}\cdot n_{s}\cdot exp(-\frac{\Delta G_{r}^{*}}{KT}),$$

where r* is the critical radius of nucleation, Δ*T* is the degree of supercooling, *T_m_* is the melting point of solid, *T* is the absolute temperature, Sigma is the surface tension, *L_m_* is the heat of melting, which refers to the heat absorbed by the system into the environment during the solid–liquid phase change reaction, *I* is the uniform nucleation rate, *D* is the nucleation rate factor affected by phase change activation energy, *P* is the nucleation rate factor affected by particle diffusion, Δ*G_a_* is the transition frequency of atoms or molecules, and $\Delta G_{r}^{*}$ is the uniform nucleation activation energy.

**Figure 6:**
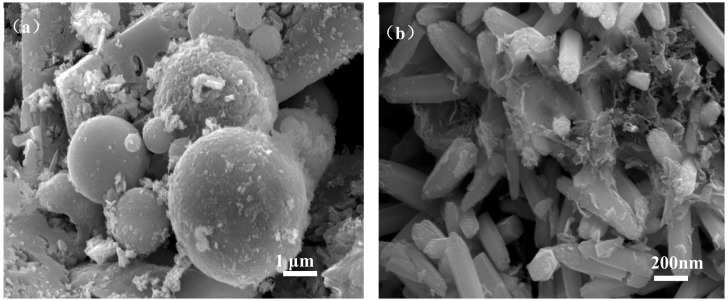

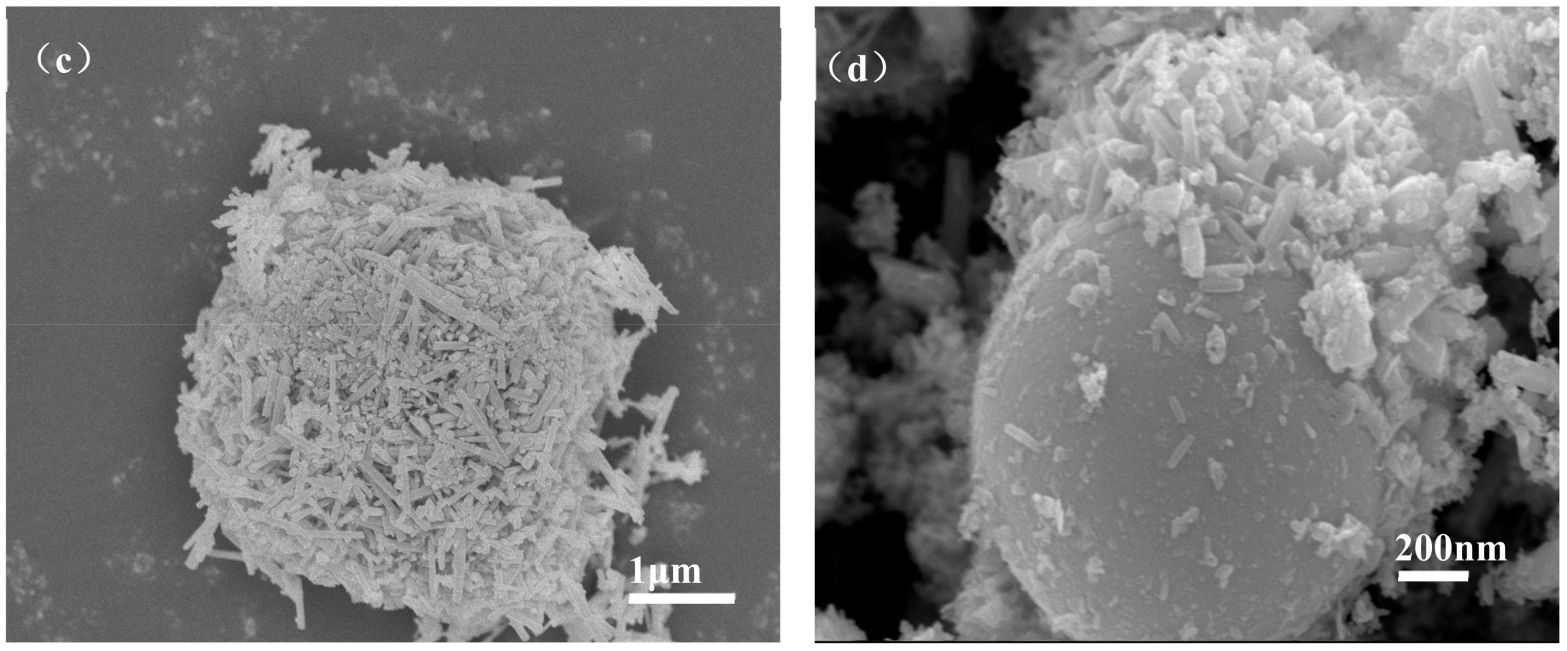
SEM images of ZCFA powder under different reaction temperatures: (**a**) 60 °C; (**b**) 70 °C; (**c**) 80 °C; (**d**) 90 °C.

Figure 6 shows the micromorphology of ZCFA composite powders at different reaction temperatures from 60 to 90 °C. It can be seen that the reaction temperature has an important effect on the morphology of the ZCFA composite. As can be seen from Figure 6, acicular or columnar ZnO appeared on the surface of CFA after coating. As the reaction temperature increased, the diameter of columnar ZnO became smaller and the length to diameter ratio increased, but it was not uniform enough. This indicates that a low temperature is conducive to the formation of ZnO with a large particle size and uniform morphology, while a high temperature is conducive to the formation of ZnO with a small particle size. In Figure 6a, the reaction temperature is low, the velocity of ion movement is low, and the precipitation particle size is coarse, about 2 microns in diameter. In Figure 6b, the reaction temperature was 70 °C, the ZnO precipitates formed were hexagonal prismatic crystals with a diameter of about 200~400 nm, arranged in “chrysanthemum” clusters. Compared with Figure 6b, when the reaction temperature increases to 80 °C (Figure 6c), the crystal diameter of ZnO decreases, which is about less than 100 nm, and the length to diameter ratio increases. ZnO crystal is uniform in shape and belongs to the hexagonal wurtzite structure. The section is hexagonal in shape. The ZnO is no longer erect but horizontal on the surface of CFA. This irregular arrangement increases the surface roughness and particle size of the CFA, so the prepared ZCFA powder has no exposed area. When the reaction temperature is 90 °C, the ions movement rate is fast, the reaction process will be completed in a very short time, the high kinetic energy between the precursor particles increases the probability of collision and tends to form aggregates, resulting in uneven precipitation coating.

**Figure 7:**
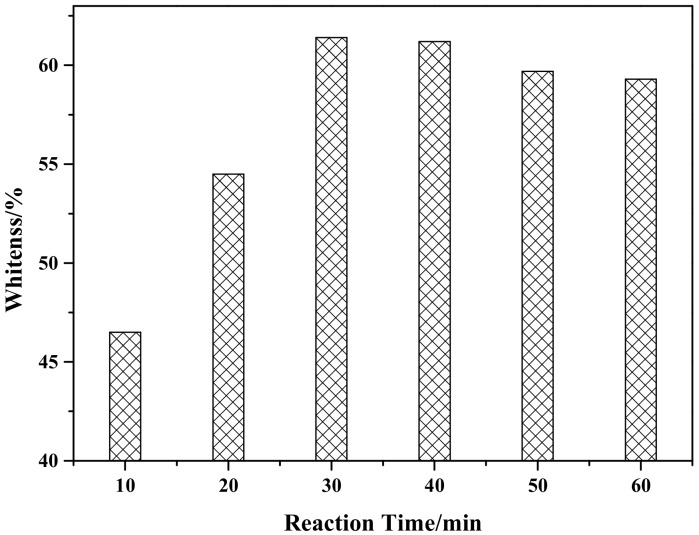
The effect of reaction time on whiteness of ZCFA powder.

Figure 7 shows the effect of reaction time on the whiteness of ZCFA composite powders. According to Figure 7, when the reaction time is 30 min, ZCFA has the highest whiteness value. As the reaction time increased, the whiteness value of the composite powder showed a decreasing trend, which was because the precipitation was not fully formed when the reaction time was 10 min. When the reaction time is 30 min, surface adsorption quantity of CFA and stripping quantity reached dynamic equilibrium. When the reaction time is longer than 30 min, ZnO particles on the surface of CFA began to fall off for mechanical force, therefore the optimum reaction time is chosen to be 30 min.

**Figure 8:**
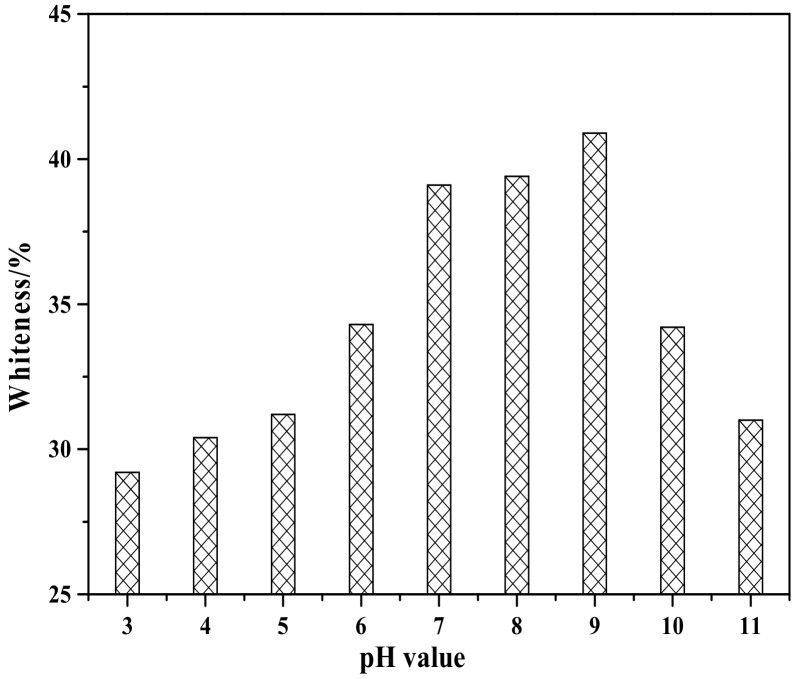
Whiteness of ZCFA powder at different pH value.

**Figure 9:**
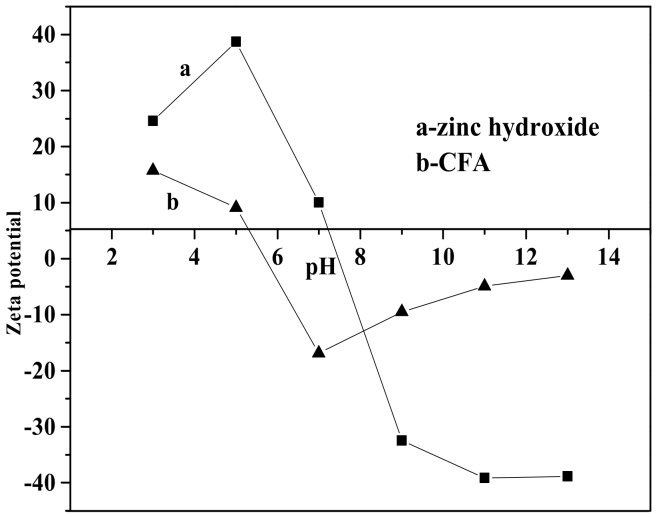
Zeta potential of zinc hydroxide and calcined fly ash (CFA) at different pH values.

Figure 8 shows the effect of pH value on the whiteness of ZCFA composite powders. It can be seen from Figure 8 that the whiteness of ZCFA powder is low in the environment of strong acid and strong alkali (pH < 5 and pH > 9). The reasons are as follows: (1) Zn(OH)_2_ belongs to amphoteric hydroxide, which is easy to dissolve in strong acid and strong alkali. When dissolved in strong acid, it forms zinc salt, and when dissolved in strong alkali, it forms zincate. Therefore, the coating layer formed in these two experimental conditions will reduce the whiteness of composite powders. (2) When the pH value is adjusted to acidic after the completion of dropping coating agent, it will neutralize NaOH, leading to the reduction of the ZnSO_4_ amount, thus reducing the content of Zn(OH)_2_ precipitation. Therefore, the whiteness of composite powders is not high. When pH = 9, the content of coating agent can complete the reaction, so the whiteness is relatively high. Under the environment of weak alkali (pH = 9), the concentration of OH^-^ is relatively high but could not dissolve the generated coating layer, which reached hydrolysis dynamic balance: [Zn(H_2_O)_n_]^2+^↔Zn(OH)_2_↓ + 2H^+^ + (n-2)H_2_O, and is beneficial to promote the reaction to the direction that the precipitate generated at this time. OH^-^ will decrease the particle spacing, prompting particles to get together and grow up, resulting the increase of the whiteness. Therefore, the optimal pH value is 9. Figure 9 shows zeta potential diagrams of CFA and zinc hydroxide at different pH values. As can be seen that the isoelectric point of Zn(OH)_2_ is 7.5, the net value of particle surface charge is zero, the farther the distance isoelectric point is, the smaller the particle size is. When the particle size is too small, it tends to form aggregate because of the large surface energy between the particles, which is not suitable for a coating layer, so it is better to prepare the Zn(OH)_2_ at pH = 9. Under this environment, the zeta potential value of Zn(OH)_2_ is −32.4, which can be relatively stable. At this time, CFA also carries a negative charge, and there is electrostatic repulsion between the two, that is, they will not be bound together by physical adsorption, but there is a chemical bond force stronger than the van der Waals force.

**Figure 10:**
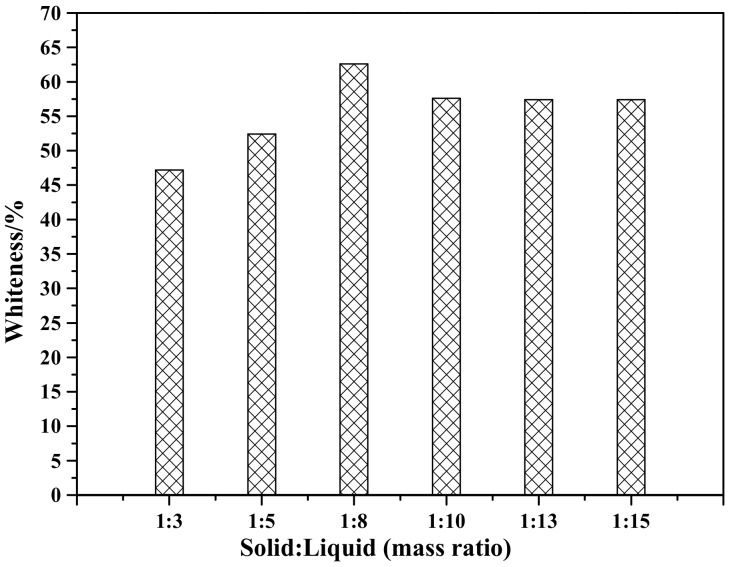
Whiteness of ZCFA powder at different solid-to-liquid ratios.

Figure 10 shows the effect of solid-to-liquid ratio on the whiteness of ZCFA composite powders. As can be seen, the whiteness reaches the highest when the solid-to-liquid ratio is 1:8.

**Figure 11:**
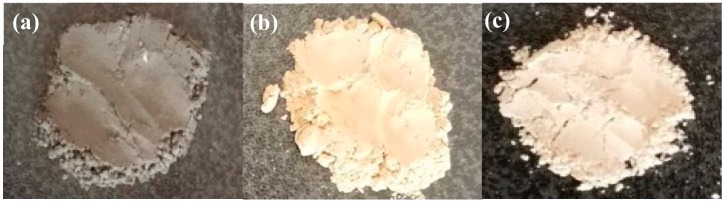
Whiteness pictures of (**a**) fly ash (FA), (**b**) CFA, and (**c**) ZCFA powders.

In conclusion, when the coating amount was 90%, the pH was 9, solid-to-liquid ratio was 1:8, the reaction temperature was 80 °C, the concentration of NaOH was 0.30 mol/L, the dropping speed was 2 mL/min, the concentration of ZnSO_4_ was 0.15 mol/L, the dropping speed was 2 mL/min, the reaction time was 30 min, and the whiteness of the prepared ZCFA composite powder was increased from 27.0 to 62.6% (Figure 11).

### 3.2. Morphology of ZCFA

**Figure 12:**
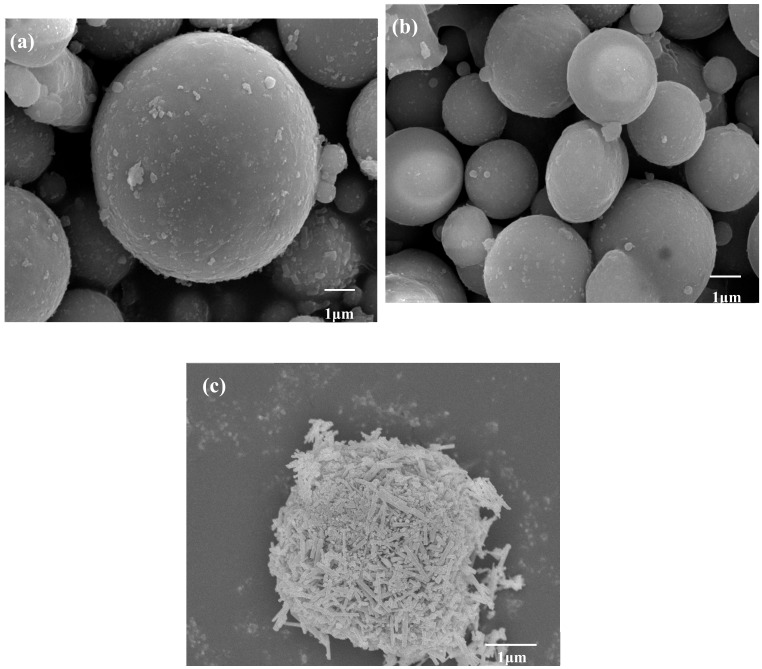
SEM pictures of (**a**) FA, (**b**) CFA, and (**c**) ZCFA powders.

Figure 12 shows the micromorphology of FA, CFA, and ZCFA powders. It can be seen from Figure 12a that FA has a regular spherical structure with uneven particle size and a smooth surface. A few irregular carbon particles adhere to the surface of FA. Due to the dark color of carbon particles, the whiteness of FA is relatively low. By comparison with Figure 12b, the content of carbon decreases after calcination, but the sphericity does not change, indicating that the physical structure of FA will not be destroyed after calcinating at 815 °C for 2 h. It can be seen from Figure 12c that the surface coating layer has a needle shape, the diameter of zinc oxide crystal is about less than 100 nm. The CFA has almost no exposed area and the coating of ZnO increases its surface roughness, which is beneficial to filling in PA6.

### 3.3. XRD Analysis

**Figure 13:**
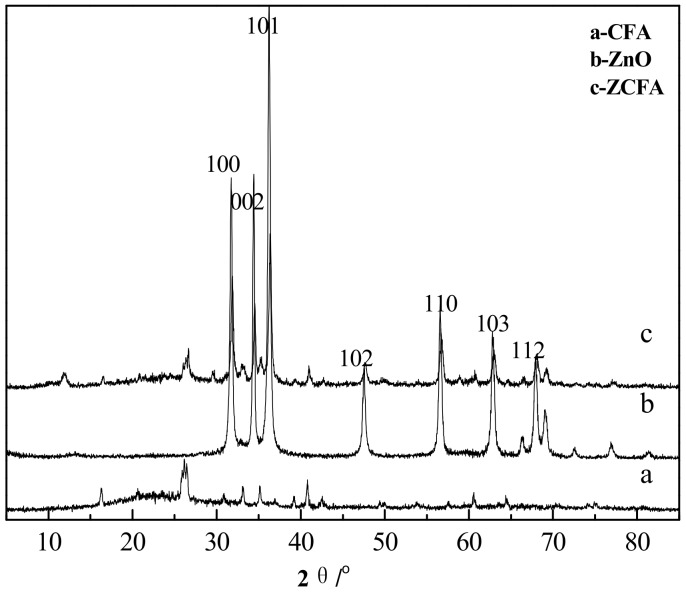
XRD spectrum of CFA, ZnO, and ZCFA powders.

Figure 13 is the XRD spectrum of CFA, ZnO, and ZCFA powders. The pattern of ZCFA peaks is mostly similar with the CFA peaks. New peaks appeared in ZCFA, belonging to needle ZnO (No.361451). The relative peaks change, and the small shift to the higher diffraction angles indicates that the CFA crystalline structure was compressed after coating with ZnO. The average crystallite size of ZnO (46 nm) on the surface of CFA can be calculated by the Scherrer equation (D = Kλ/(βcosθ), where β = 0.00305 is the full width at half maxima (FWHM) of the sample and θ is the diffracting angle, K = 0.94 is a coefficient, and λ = 0.15418 nm is the X-ray wavelength [21]. 

### 3.4. FTIR Analysis

**Figure 14:**
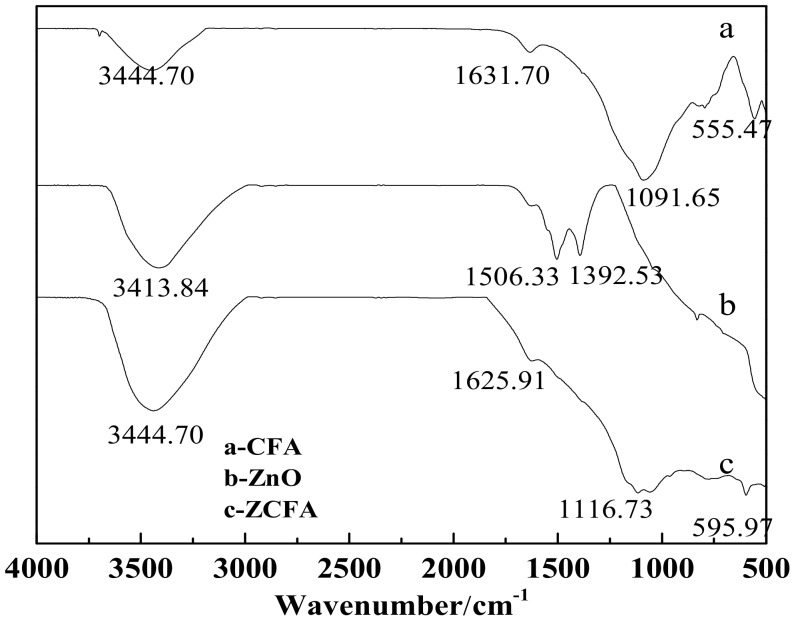
FTIR of CFA, ZnO, and ZCFA powders at 500–4000 cm^-1.^

**Figure 15:**
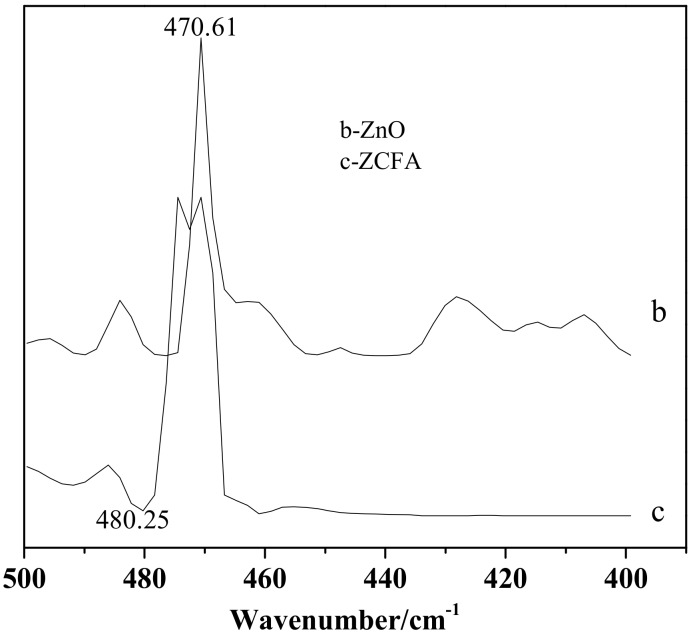
FTIR of ZnO and ZCFA powders at 400–500 cm^-1^.

Figure 14 is the FTIR of CFA, ZnO, and ZCFA powders at 500–4000cm^-1^. Figure 15 is the FTIR of ZnO and ZCFA powders at 400–500 cm^-1^. The band at 470.61 cm^-1^ in pure ZnO was assigned Zn–O stretching. The band stretches at the 3413.84 cm^−1^ and that at 1100–1600 cm^−1^ are corresponded to hydroxyl bonding of Zn–OH (Figure 14b) [22].

The adsorption band at 500–520 cm^-1^ for CFA (Figure 14a) was associated with Al–O stretching vibration [23]. The band at 555.47 cm^-1^ was ascribed to the Si–O bending vibration of CFA [23]. The bands at 1091.65 cm^-1^ corresponded with the Si–O–Si asymmetric stretching vibration adsorption peak [24]. The band at 3440.39 cm^-1^ was attributed to the O–H stretching vibration characteristic adsorption peak [24]. The band at 1631.7 cm^-1^ was associated with the O–H bending vibration of CFA [23]. 

Comparing FTIR spectrum of ZCFA with CFA and ZnO, it can be observed that the O–H bending vibration peak at the 1631.7 cm^-1^ has shifted to 1625.91 cm^-1^, which is due to the appearance of Zn^2+^ replacing some of the original Si^4+^, and the atomic number of cation increases, making the adsorption band shift to the low frequency. There are no peaks at 3690 cm^-1^ in Figure 14b,c, that is, there is no typical hydrogen and oxygen bond, indicating that the precursor Zn(OH)_2_ has been completely decomposed into ZnO. The band at 1091.65 cm^-1^ shifted to 1116.73 cm^-1^, the peak broadened and the peak intensity decreased, the band at 1631.7 cm^-1^ shifted to 1625.91 cm^-1^ and the intensity decreased, the new peak of 470.61 cm^−1^ appeared in the ZCFA, all of these illustrating that Si–O–Zn–OH bonds were formed in ZCFA, which is beneficial for the organic modification of ZCFA in the later experiment.

### 3.5. Specific Surface Area and Pore Characteristics

Table 1 shows the specific surface areas and pore characteristics of FA, CFA, and ZCFA. It can be observed that the BET specific surface area of FA decreased from 5.80 to 4.51 m^2^/g after calcination, which was attributed to the removal of carbon. The BET specific surface area of CFA increased from 5.80 to 14.61 m^2^/g after coating with ZnO, the volume of the pore increased from 0.0112 to 0.0324 cm^3^/g. The reason for this is that the gaps which are not completely filled by the coated particles become pore like structures.

## 4. Conclusions

The whiteness of the ZCFA powders was affected by different experimental conditions, such as pH value, reaction temperature, coating amount, solid-to-liquid ratio, dropping speed, reaction time, and the concentration of NaOH and ZnSO_4_. The results showed that the optimum conditions were as follows: (1) Coating amount and pH value were 90% and 9, respectively; (2) reaction temperature and time were 80 °C for 30 min; (3) solid-to-liquid ratio was 1:8; (4) the concentration of NaOH and ZnSO_4_ was 0.30 and 0.15 mol/L, respectively. (5) The dropping speed of NaOH and ZnSO_4_ was 2 mL/min, respectively. SEM, BET, XRD, and FTIR analysis showed that ZCFA powders were synthesized under these optimum conditions. The surface of CFA was coated with needle ZnO with a particle size of 46 nm from SEM analysis and Scherrer equation. The ZCFA powders prepared under the optimum preparation conditions showed high whiteness that was increased from 27.0 to 62.6%. Si–O–Zn–OH bonds were formed from FTIR analysis, which is beneficial to the organic modification of ZCFA in the later experiment. 

## Figures and Tables

**Table 1:** Specific surface areas and pore characteristics of FA, CFA, and ZCFA powders.

Samples	S_BET_ (m^2^/g)	V_total_ (cm^3^/g)	V_meso_ (cm^3^/g)	V_mac_ (cm^3^/g)	D (nm)
FA	5.80	0.0113	0.0112	0.0001	4.75
CFA	4.51	0.0090	0.0089	0.0001	4.86
ZCFA	14.61	0.0327	0.0324	0.0003	5.86
